# Highly efficient generation of knock-in transgenic medaka by CRISPR/Cas9-mediated genome engineering

**DOI:** 10.1186/s40851-017-0086-3

**Published:** 2018-02-05

**Authors:** Ikuko Watakabe, Hisashi Hashimoto, Yukiko Kimura, Saori Yokoi, Kiyoshi Naruse, Shin-ichi Higashijima

**Affiliations:** 1 National Institutes of Natural Sciences, Okazaki Institute for Integrative Bioscience, National Institute for Basic Biology, Higashiyama 5-1, Myodaiji, Okazaki, Aichi 444-8787 Japan; 2 Bioscience and Biotechnology Center, Nagoya University, Nagoya, Aichi 464-8601 Japan; 3 National Institutes of Natural Sciences, National Institute for Basic Biology, Okazaki, Aichi 444-8585 Japan; 4 Department of Basic Biology, School of Life Science, Graduate University for Advanced Studies (SOKENDAI), Okazaki, Aichi 444-8787 Japan; 5 Present address: Faculty of Pharmaceutical Sciences, Hokkaido University, Sapporo, Hokkaido 060-0812 Japan

**Keywords:** Medaka, Transgenic, CRISPR/Cas9, Knock-in

## Abstract

**Background:**

Medaka (*Oryzias latipes*) is a popular animal model used in vertebrate genetic analysis. Recently, an efficient (~ 30%) knock-in system via non-homologous end joining (NHEJ) was established in zebrafish using the CRISPR/Cas9 system. If the same technique were applicable in medaka, it would greatly expand the usefulness of this model organism. The question of the applicability of CRISPR/Cas9 in medaka, however, has yet to be addressed.

**Results:**

We report the highly efficient generation of knock-in transgenic medaka via non-homologous end joining (NHEJ). Donor plasmid containing a heat-shock promoter and a reporter gene was co-injected with a short guide RNA (sgRNA) targeted for genome digestion, an sgRNA targeted for donor plasmid digestion, and Cas9 mRNA. Broad transgene expression in the expression domain of a target gene was observed in approximately 25% of injected embryos. By raising these animals, we established stable knock-in transgenic fish with several different constructs for five genetic loci, obtaining transgenic founders at efficiencies of > 50% for all five loci. Further, we show that the method is useful for obtaining mutant alleles. In the experiments where transgene integrations were targeted between the transcription start site and the initiation methionine, the resultant transgenic fish became mutant alleles.

**Conclusion:**

With its simplicity, design flexibility, and high efficiency, we propose that CRISPR/Cas9-mediated knock-in via NHEJ will become a standard method for the generation of transgenic and mutant medaka.

**Electronic supplementary material:**

The online version of this article (10.1186/s40851-017-0086-3) contains supplementary material, which is available to authorized users.

## Background

Medaka (*Oryzias latipes*) is a small freshwater teleost species. Similar to zebrafish (*Danio rerio*), medaka is a popular animal model for vertebrate genetic analysis and offers many advantages, including the availability of highly polymorphic inbred strains that can be effectively used for genetic mapping [1–3].

Transgenic animals with reporter expression in specific tissues or cell types are valuable tools, and many transgenic strains have been generated in medaka [1, 4]. Traditional methods for the generation of transgenic medaka, however, require promoter/enhancer hunting or bacterial artificial chromosome (BAC) modification, both of which involve time-consuming steps. Recently, the targeted knock-in of a reporter construct via a homology-dependent DNA repair was shown to work well in medaka using the CRISPR/Cas9 system [5]. Although the technique is ideal for precise knock-in, it also requires time-consuming molecular cloning steps for the construction of a donor plasmid. In zebrafish, an efficient (~ 30%) knock-in system via non-homologous end joining (NHEJ) has been established [6]. In this method, the co-injection of donor plasmid, short guide RNAs (sgRNAs), and Cas9 mRNA lead to the concurrent digestion of the genomic DNA and the donor plasmid, resulting in the incorporation of the donor plasmid into the genome. The technique does not require molecular cloning steps for the construction of a donor plasmid, and is now becoming standard for the generation of transgenic fish with reporter gene expression in a specific tissue. The technique has also been shown to be useful for the generation of mutant alleles [7]. If the same technique were shown to be applicable in medaka, it would greatly expand the usefulness of this model organism. To date, however, this question has remained unresolved.

We show that reporter constructs consisting of a medaka heat shock promoter (expected to work as a minimum promoter) and reporter genes integrated into the aimed genomic loci with high frequency via CRISPR/Cas9-mediated NHEJ; more than 50% of raised animals became transgenic founders. We further show that integrations can lead to the disruption of a gene when the integration was targeted between the transcription start site and the initiation methionine. Given its simplicity, design flexibility, and high efficiency, we propose that CRISPR/Cas9-mediated knock-in via NHEJ will become a standard method for the generation of transgenic and mutant medaka.

## Methods

### Fish care and strains

Medaka adults, embryos, and larvae were maintained at 25–28 °C. All procedures were performed in compliance with the guidelines approved by the animal care and use committees of the National Institutes of Natural Sciences and Nagoya University. Animals were staged by days post fertilization (dpf). The parental strain for the generation of all transgenic fish was d-rR. The genetic background was Nagoya for *ml-3* (*sox5*), orange-red variety for *lf-2* (*pax7a*)*,* and d-rR for *pnp4a.*

### Construction of donor DNA for knock-in

Tbait (GGCTGCTGTCAGGGAGCTCATGG) sequence [6] was used as a bait sequence in donor plasmids. Two types of donor plasmids were used in this study: Tbait-hs-loxP-RFP-loxP-GFP and Tbait-hs-GFP. The hs represents the 0.8 kb sequence from the medaka hsp70 promoter (the *hsp70.1* gene; [8]). The sequence was amplified by polymerase chain reaction (PCR) with primers: AGCTGCGTCACGTGGTCCCG (forward) and TGCTTTGTGCTGTAAAGACGC (reverse). Except for the usage of the medaka hs promoter, the constituents of Tbait-hs-loxP-RFP-loxP-GFP and Tbait-hs-GFP plasmids are essentially as described in [6] and [7].

### Construction of zhspa8:Cre and generation of Tg[zhspa8:Cre-mCherry-NLS] transgenic fish

Zebrafish hspa8 promoter, approximately 2.6 kb in length [9, 10], was used to express Cre-mCherry-NLS [11] ubiquitously in early embryos. The zhsp8 promoter, Cre-mCherry-NLS, and bovine growth hormone (BGH) polyA sequences were placed in this order in the Tol2-based vector, pT2KXIGΔin [12]. Microinjection of Tol2-based plasmid DNA into medaka embryos was performed as was done in zebrafish [12].

### Preparation of sgRNAs

Template DNA for sgRNA synthesize was PCR-amplified from pDR274 [13] with the forward primer, ATTTAGGTGACACTATAgaxxxxxxxxxxxxxxxxxxGTTTTAGAGCTAGAAATAGC (for SP6 polymerase) or TAATACGACTCACTATAggxxxxxxxxxxxxxxxxxxGTTTTAGAGCTAGAAATAGC (for T7 polymerase), and the reverse primer, AAAAGCACCGACTCGGTGCC. The lowercase letters correspond to genome-targeting sequences (either 19 or 20 mer) in sgRNAs. The genome-targeting sequences in sgRNAs used in this study are shown in Table 1. After PCR amplification with Prime Star Taq polymerase (Takara, Otsu, Japan), PCR product was purified using a QIAquick PCR Purification Kit (Qiagen, Hilden, Germany). Template DNA thus obtained was used for the in vitro transcription of sgRNAs using a MAXIscript T7 kit (Life Technologies, Carlsbad, USA). pCS2-hSpCas9 (a gift from M. Kinoshita and F. Zhang; [14]) was digested with NotI, and Cas9 mRNA was transcribed using an mMESSAGEmMACHINE SP6 kit (Life Technologies). sgRNAs and Cas9 mRNA were purified using an RNeasy Mini kit (Qiagen).

**Table 1:**
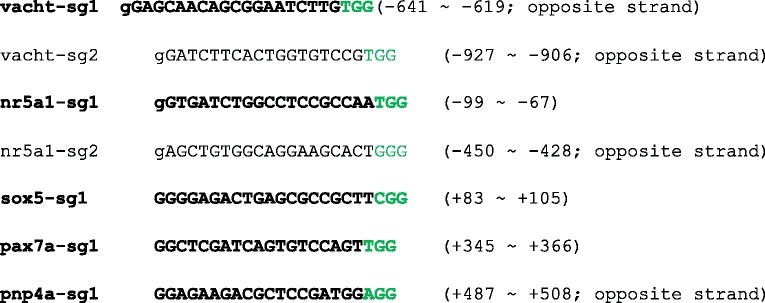
DNA sequences for the corresponding sgRNAs

The protospacer-adjacent motif (PAM) sequence is labeled in green. The bold letters indicate that the corresponding sgRNAs were used to generate knock-in transgenic fish. The lowercase letter, g, at the beginning of the sequences indicates that the corresponding G is not present in the genome (mismatch). The mismatches derive from the demand that the first letter ought to be G for efficient in vitro transcription by T7 or SP6 polymerase. The numerals shown at the right indicate the locations of the corresponding sequences with respect to the prospective transcription start sites (the 5’end of the longest cDNA) for each gene

For the *vacht* and *nr5a1* genes, we tested two sgRNAs. The sgRNA that yielded best results in F0 expression assay was chosen to generate stable transgenic fish (Table 1). For the *sox5*, *pax7a*, and *pnp4a* genes, we tested just one sgRNA for each gene (Table 1).

### Microinjection for knock-in

sgRNAs and Cas9 mRNA were co-injected into one-cell stage medaka embryos with Qiagen miniprep (Qiagen) purified donor DNA. Each embryo was injected with a solution containing ~ 9 ng/μl of sgRNA for digesting Tbait, ~ 18 ng/μl of sgRNA for digesting genome DNA, ~ 200 ng/μl of Cas9 mRNA, and ~ 9 ng/μl of donor plasmid. Injection volume was adjusted such that approximately 30–75% of injected embryos were dead within one week after injection (Table 2).

**Table 2:** Results of microinjections and screening of transgenic founders

	injected	survived at 7dpf	good expression	survived to adult	positive founder
vacht sg1	84	20	11	10	5 / 10 (50%)
nr5a1 sg1	123	31	3	1	1 / 1 (100%)
sox5 sg1	52	25	11	10	7/ 10 (70%)
pax7a sg1	75	32	9	7	6 / 7 (85.7%)
pnp4a sg1	237	69^*^	13	6	3 / 6 (50%)
total	571	177	47	34	23 / 34 (67.6%)

The asterisk (*) indicates the number of surviving fish at 2 dpf

### Insertion mapping

For insertion mapping, fluorescent F1 animals at 5–9 dpf were collected, and genomic DNA was extracted with standard protocols. The insertion status was examined on either the 5′ side or the 3′ side of the insertion. For example, to examine the 5′ side of the insertion, a PCR reaction was performed using a 5′ primer that was specific to each gene (upstream of the expected insertion site) and a 3′ primer that was specific to the donor plasmid (sequence within the hsp70 promoter for detecting the forward insertion, and sequence within pBluescriptSK for detecting the inverse insertion). To examine whether the tandem-array insertion in the same direction occurred, a PCR reaction was performed with the two primers within the donor plasmid.

For the *sox5* and *pax7a* transgenic fish, nucleotide sequences of PCR products were determined to examine the joint regions of the insertions.

### Imaging

Images were taken using an MVX10 microscope (Olympus, Tokyo, Japan), an MZ APO stereomicroscope (Leica, Wetzlar, Germany), and an LSM700 confocal laser-scanning microscope (Zeiss, Oberkochen, Germany).

## Results

### Strategy for the generation of knock-in medaka and generation of Tg[vacht-hs:lRl-GFP] strains

**Fig. 1:**
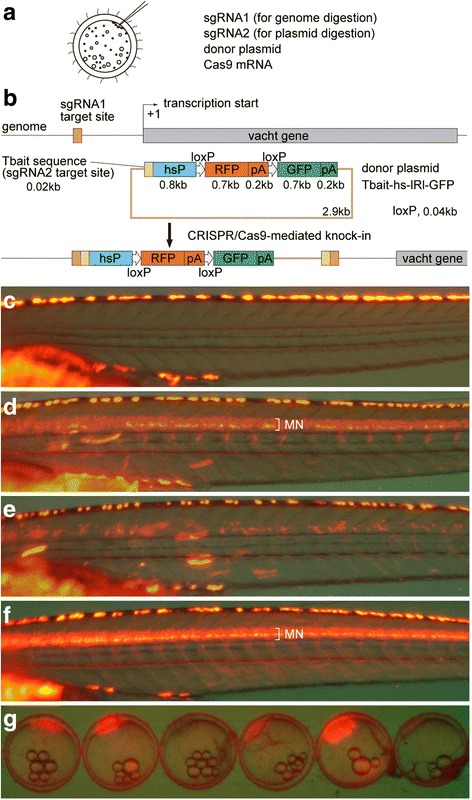
Strategy for the generation of knock-in medaka and generation of Tg[vacht-hs:lRl-GFP] strains. (**a**) For the generation of knock-in transgenic fish, sgRNA1 (for genome digestion), sgRNA2 (for plasmid digestion), donor plasmid with a bait sequence, and Cas9 mRNA are co-injected into one-cell-stage medaka embryos. (**b**) A schematic representation of the *vacht* locus (grey box) and the sgRNA target sites (orange box), and the reporter gene construct consisting of the Tbait (brown box), medaka hsp70 promoter (hsP, blue box), loxP, RFP-pA (red box), loxP, and GFP-pA (green box). After injection, the concurrent cleavage of the targeted genomic locus and the Tbait-hs-lRl-GFP reporter plasmid results in the integration of the reporter by non-homologous end joining (NHEJ). The scheme shows the forward integration of the reporter. (**c**) Lateral view (red fluorescence) of a control larva at 9dpf. Red-yellow signals in the dorsal region of the body are from the auto-fluorescence of pigment cells. (**d**) An example of an injected larva. RFP expression was present broadly in the motoneurons (MN). Animals with this kind of RFP expression were judged as having “good expression”, and raised to adulthood. (**e**) Another example of an injected larva. In this animal, RFP expression was present in the motoneurons, but the number of RFP-expressing cells is much smaller than that in (**d**). Animals with this kind of RFP expression were judged as not having “good expression”, and were not raised. (**f**) Lateral view of a Tg[vacht-hs:lRl-GFP] larva. RFP expression was present broadly in the trunk motoneurons. All of the trunk motoneurons are likely to express RFP in this animal. (**g**) Maternal expression of RFP in early embryos in the Tg[vacht-hs:lRl-GFP] line. The expression levels of RFP are variable among embryos. The embryos were obtained from a single mother

For NHEJ-mediated knock-in in medaka, we employed an experimental scheme previously established by our group in zebrafish [6]. Briefly, we co-injected sgRNA1 (for genome digestion), sgRNA2 (for plasmid digestion), donor plasmid, and Cas9 mRNA into one-cell-stage medaka embryos (Fig. 1a). The *vacht* (*vesicular acetylcholine transporter*; also called *slc18a3*) gene, which is known to be expressed in cholinergic neurons (motoneurons, primarily), was chosen as an initial target. The donor plasmid (Tbait-hs-lRl-GFP) contains a bait sequence (Tbait; [6]) upstream of the insertion cassette for sgRNA2-guided DNA cleavage. This bait sequence was selected because the corresponding sgRNA (sgT) appears to have no off-target site in the medaka genome (Additional file 1: Table S1). Tbait is followed by a medaka hsp70 promoter, which we expect to work as a minimal promoter (Fig. 1b). In this study, we extracted a 0.8 kb sequence of the medaka hsp70.1 promoter (see Methods) for this purpose. The hsp70 promoter is followed by the loxP-RFP-loxP-GFP (lRl-GFP) sequence (Fig. 1b). Without application of Cre recombinase, RFP will be expressed as a reporter gene. The target site for genome digestion was set upstream of the prospective transcriptional-start site of *vacht* (Fig. 1b; the sequences used are shown in Table 1). Concurrent digestion of the genome (guided by sgRNA1) and the plasmid DNA (guided by sgRNA2) with Cas9 would result in the integration of the donor plasmid into the genome via an NHEJ (Fig. 1b).

We tested two sgRNAs (vacht-sg1 and vacht-sg2; Table 1). Injected animals were investigated for their RFP expression around the hatching stage. In the case of vacht-sg1, we found animals that showed wide-spread RFP expression in the trunk motoneurons (Fig. 1d; 1c is a control animal) in 55% (11 of 20) of the animals (Table 2). The remaining 45% of the animals either showed sparse RFP expression in the motoneurons (Fig. 1e) or no expression. In the zebrafish experiments, there was a strong correlation between the expression levels of a reporter gene in injected animals and the probability of becoming transgenic founders. Animals that had good reporter gene expression had a high probability of becoming positive founders [6]. Thus, we raised only those animals that were considered to have “good expression” (Fig. 1d; Table 2). The raised animals were crossed to wild-type to examine if they would produce fluorescent offspring. Among 10 fish screened, five produced larvae with RFP expression in the motoneurons (Fig. 1f; Table 2). The expression patterns of RFP in these Tg [vacht-hs:lRl-GFP] transgenic fish were similar among progenies from different founders. We investigated the insertion sites of each line by PCR, and found that, in all cases, the transgene was integrated around the expected site in the genome. As was seen in zebrafish, both forward-direction integration and reverse-direction integration were observed. Moreover, in some cases multiple copies of donor plasmid were integrated (Additional file 2: Table S2).

The medaka hsp70 promoter employed in this study showed activity in female germ cells. Embryos produced from transgenic females showed ubiquitous red fluorescence due to the maternal effect (Fig. 1g). Expression levels were variable even among progenies from the same female (Fig. 1g). This maternally-derived fluorescence became negligible at around 3 dpf, and thus did not represent a major problem for observation of RFP-labeled motoneurons at later stages. The maternally derived fluorescence was also observed in other lines generated in this study.

### Conversion of RFP transgenic fish to GFP transgenic fish by crossing

The reporter sequence (loxP-RFP-loxP-GFP) in the Tg[vacht-hs:lRl-GFP] fish described above was aimed such that RFP expression could be converted to GFP expression by the application of Cre. To conveniently change RFP transgenic fish to GFP transgenic fish, we generated transgenic medaka that ubiquitously express Cre-mCherry-NLS fusion protein [11] in early embryos. For this purpose, we used the zebrafish hspa8 promoter, which is known to drive gene expression ubiquitously in early zebrafish embryos [9, 10].

**Fig. 2:**
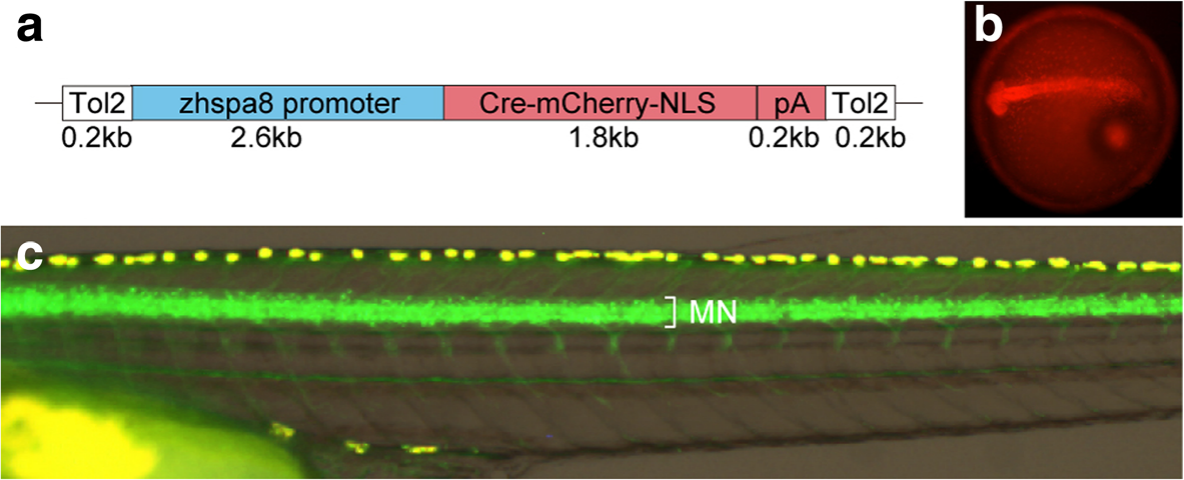
Generation of Tg[zhspa8:Cre-mCherry-NLS] transgenic fish and conversion of RFP transgenic fish to GFP transgenic fish. (**a**) A schematic of the zhspa8:Cre-mCherry-NLS plasmid. The plasmid consists of the Tol2 left arm (white box), zebrafish hspa8 promoter (zhspa8; blue box), Cre-mCherry-NLS and bovine growth hormone (BGH) polyA signal (red box), and Tol2 right arm (white box). (**b**) A 1dpf embryo of the Tg[hspa8:Cre-mCherry-NLS] transgenic fish. Red fluorescence derived from Cre-mCherry-NLS is present ubiquitously in the embryonic body. (**c**) A 9dpf larva derived from the crossing between Tg[vacht-hs:lRl-GFP] and Tg[hspa8:Cre-mCherry-NLS]. GFP instead of RFP is expressed in the trunk motoneurons (MN)

Tg[zhsp8:Cre-mCherry-NLS] transgenic medaka was generated by a Tol2-based transgenic method (Fig. 2a). Transgenic embryos of this line expressed Cre-mCherry-NLS proteins ubiquitously in the early stages (Fig. 2b). We crossed this Tg[zhsp8:Cre-mCherry-NLS] fish to Tg[vacht-hs:lRl-GFP] transgenic fish. As expected, we obtained larvae that expressed GFP instead of RFP in the motoneurons (Fig. 2c). The GFP-expressing animals were raised to adulthoods, and crossed to wild-type fish. Approximately one-half of the fish expressed GFP in the motoneurons; no RFP-expressing fish were obtained. These results indicate that RFP transgenic fish can be genetically transformed to GFP transgenic fish (Tg[vacht-hs:GFP]) by crossing alone.

### Generation of Tg[nr5a1-hs:lRl-GFP] and Tg[nr5a1-hs:GFP] strains

**Fig. 3:**
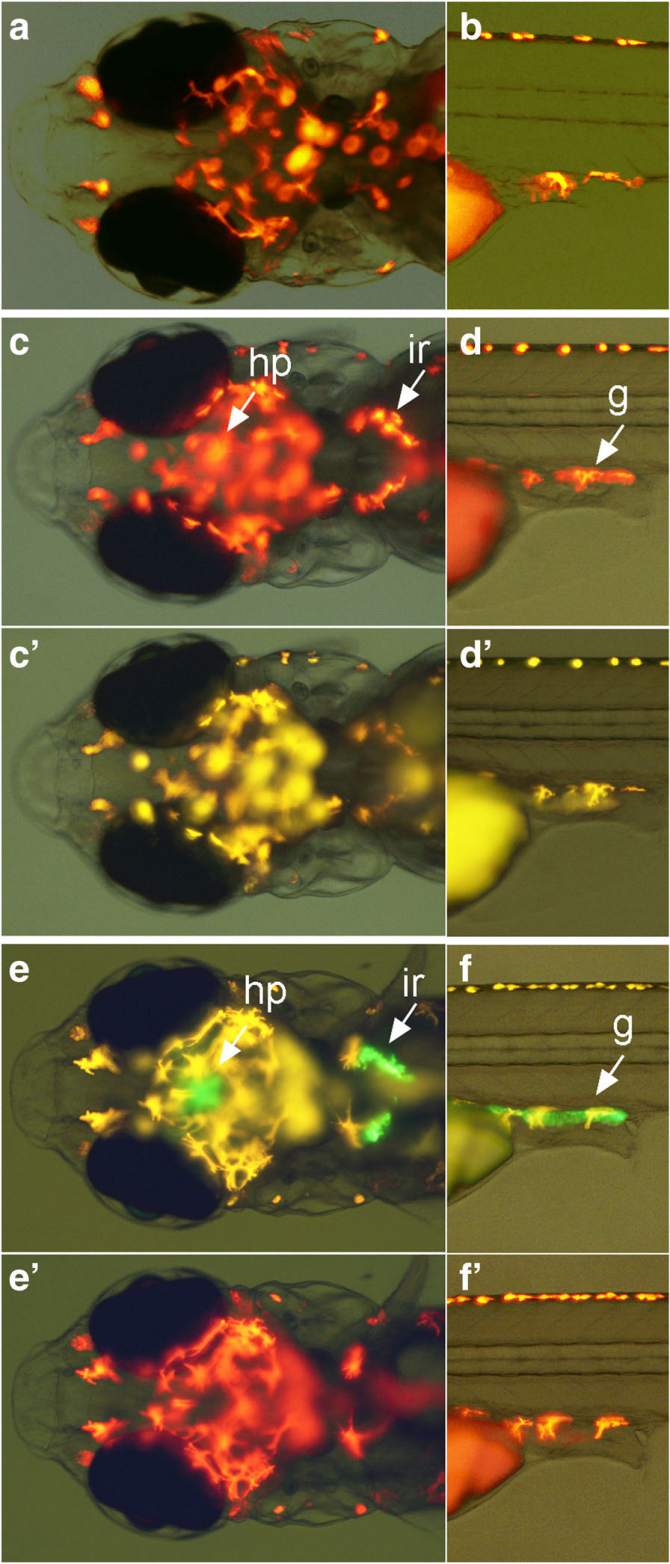
Generation of Tg[nr5a1-hs:lRl-GFP] and Tg[nr5a1-hs:GFP] transgenic fish. (**a**, **b**) Dorsal view of the head region (**a**) and lateral view of the trunk region (**b**) of a control larva at 9dpf with an RFP filter set. Red signals are from the auto-fluorescence of pigment cells. (**c**, **d**) Tg[nr5a1-hs:lRl-GFP] transgenic fish viewed with an RFP filter set. RFP is expressed in cells in the hypothalamus (hp), interrenal gland (ir), and gonad (g). Note that the red signals are not auto-fluorescence, as the corresponding signals are absent in a control larva (**a** and **b**). (c', d') Tg[nr5a1-hs:lRl-GFP] transgenic fish viewed with a GFP filter set. No GFP expression is present. (**e**, **f**) Tg[nr5a1-hs:GFP] transgenic fish with a GFP filter set. GFP is expressed in cells in the hypothalamus (hp), interrenal gland (ir), and gonad (g). Yellow signals are from the auto-fluorescence of pigment cells. (e', f') Tg[nr5a1-hs:GFP] transgenic fish viewed with an RFP filter set. No RFP expression is present

Next, we chose the *nr5a1* (also called *ftz-f1*) gene, and examined if the same technique worked for this gene. The *nr5a1* gene is known to be expressed in cells in the hypothalamus (hp), interrenal gland (ir), and gonad (g) ([15]; see also Figs. 3e and f). We tested two sgRNAs (nr5a1-sg1 and nr5a1-sg2). In both cases, the target sites were set upstream of the prospective transcription start site (Table 1), like the experiments with the *vacht* gene. We performed microinjections with the Tbait-hs-lRl-GFP (see, Fig. 1b). In the case of nr5a1-sg1, we observed animals that had RFP expressions in the expression domains of nr5a1. Compared to the experiments with the *vacht* gene, the expression of RFP in these tissues in the injected animals was more difficult to see, because strongly auto-fluorescent red pigment cells were located near the tissues where *nr5a1* was expressed (see Figs. 3a–d). We selected three animals (out of 31 survivors) that had good RFP expression in these tissues, and raised them (Table 2). Of these three fish, only one survived to adulthood. The survivor was crossed to a wild-type fish, and it turned out that the fish was a positive founder (Tg[nr5a1-hs:lRl-GFP]). RFP expression in the transgenic fish was observed in the expected tissues (hp, ir, and g in Figs. 3a–d). No GFP expression was observed in the transgenic fish (Figs. 3c' and d'). We investigated the insertion site of the line by PCR, and found that the transgene was integrated in the expected site in the forward direction as a single copy (Additional file 2: Table S2).

We then examined if RFP fish could be converted to GFP fish by crossing. A Tg[nr5a1-hs:lRl-GFP] transgenic founder was crossed to Tg[zhsp8:Cre-mCherry-NLS] fish. This resulted in the production of animals in which GFP instead of RFP was expressed in the hypothalamus (hp), interrenal gland (ir), and gonad (g) (Figs. 3e and f). The animals were raised to adulthood, and were then verified to produce GFP-expressing fish. These GFP transgenic fish did not express RFP (Figs. 3e' and f'). Thus, RFP transgenic fish were converted to GFP transgenic fish, resulting in the establishment of Tg[nr5a1-hs:GFP].

### Generation of mutant alleles for *pax7a* and *sox5* genes by NHEJ-mediated knock-in

In the knock-in experiments for the *vacht* and *nr5a1* genes, we did not aim to disrupt gene functions. The insertion sites were set upstream of the genes, not in the exons. In zebrafish studies, the NHEJ-mediated knock-in technique has also shown to be effective for obtaining mutant alleles by inserting the transgene into exons [7]. This led us to examine whether this is also the case in medaka.

**Fig. 4:**
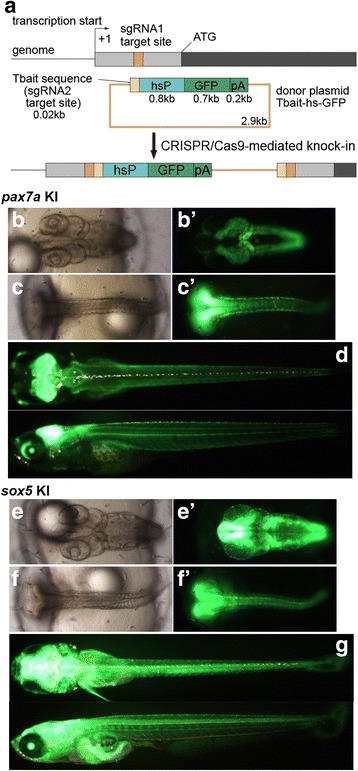
Generation of Tg[pax7a-hs:GFP] and Tg[sox5-hs:GFP] fish. (**a**) Schematic of the knock-in strategies. The sgRNAs for *pax7a* or *sox5* were designed to target a sequence upstream of the initiation methionine that corresponds to the 5′ untranslated region. (**b–d**) Tg[pax7a-hs:GFP]. (**e–g**) Tg[sox5-hs:GFP]. (**b, b', c, c', e, e', f, f'**) Dorsal views of the head (**b, b', e, e'**) and the trunk (**c, c', f, f'**) at 2 dpf in the bright field (**b, c, e, f**) and in fluorescence (**b', c', e', f'**). (**d, g**) Dorsal (upper) and lateral (lower) views at 9 dpf. In Tg[pax7a-hs:GFP] embryos, GFP is expressed in the tectum, hindbrain, anterior neural tube (**b'**), and muscles (**c'**). These fluorescent signals are maintained in the hatchlings (**d**). In Tg[sox5-hs:GFP] embryos, GFP is expressed in a range of the central nervous system (CNS) from the forebrain to hindbrain (**e'**), and in neural tubes and the premigratory neural crest in a dotted manner (**f'**). At 9 dpf, additional fluorescent signals are observed in the pectoral fins, olfactory bulbs, and presumable xanthophore progenitors on the dorsal body surface (**g**; see also Additional file 3: Fig. S1C–D)

*pax7a* (*paired*-type homeobox) and *sox5* (*sry*-related high-mobility-group box) were chosen as initial target genes, because mutations of the genes lead to skin pigmentation defects [16, 17], and the phenotypes can be identified easily by the absence or increase of pigment cells. We used Tbait-hs-GFP as a donor plasmid (Fig. 4a). The target sites were set between the transcription start site and the first methionine (Fig.4a and Table 1). We performed microinjection, and with high probability, obtained animals that had broad GFP expression in the expression domains of *pax7a* (tectum, hindbrain, etc.; see also Fig. 4b–d) or *sox5* (CNS from the forebrain to hindbrain, dorsal neural tube, etc.; see also Fig. 4e–g). We raised those animals that were considered to exhibit good expression (Table 2), and crossed them to wild-type fish. We obtained 6 *pax7a*-transgenic founders out of the seven fish raised (85.7%). The expression patterns of GFP were similar among the progenies of different *pax7a*-transgenic founders. Examples are shown in Fig. 4b–d. The fluorescent signals in the progenies of these founders mimic the endogenous *pax7a* expression, including the tectum, hindbrain, anterior neural tube, somites and pre-migratory neural crest (Fig. 4b–d), and presumable pigment cell precursors on the body surface (progenitors of xanthophore and leucophore) (see also Additional file 3: Figure S1A and B), as previously reported [16]. We also obtained seven *sox5*-trangenic founders out of the 10 raised fish (70%). The expression patterns of GFP were similar among the progenies of different *sox5*-transgenic founders, and mimic the endogenous *sox5* expression in a range of the central nervous system (CNS) from the forebrain to hindbrain, dorsal neural tube (Fig. 4e–g, see also Additional file 3: Figure S1C), and a portion of pre-migratory neural crest (Fig. 4f') and presumable xanthophore precursors (Fig. 4g, see also Additional file 3: Figure S1D). The analyses of the insertion status with PCR revealed that, except for one case, the transgene was integrated at the expected location with several forms: forward, reverse, and multiple copies (Additional file 2: Table S2). In one case, PCR analyses failed to detect amplicons on both of the 5′ and 3′ sides (#5 strain for *sox5*; Additional file 2 Table S2). In this transgenic fish, large deletions that exceeded the locations of 5′- and 3′-gene specific primers might have been introduced in the genomic DNA when the integration occurred. To reveal the sequences of the joint regions of each transgenic strain, we performed sequencing experiments for the PCR products that spanned the genomic DNA and the donor DNA (Additional file 2: Table S2). The results, which are presented in Additional file 4: Figure S2, show that indels were frequently introduced in the joint region, as was seen in the previous study [18].

**Fig. 5:**
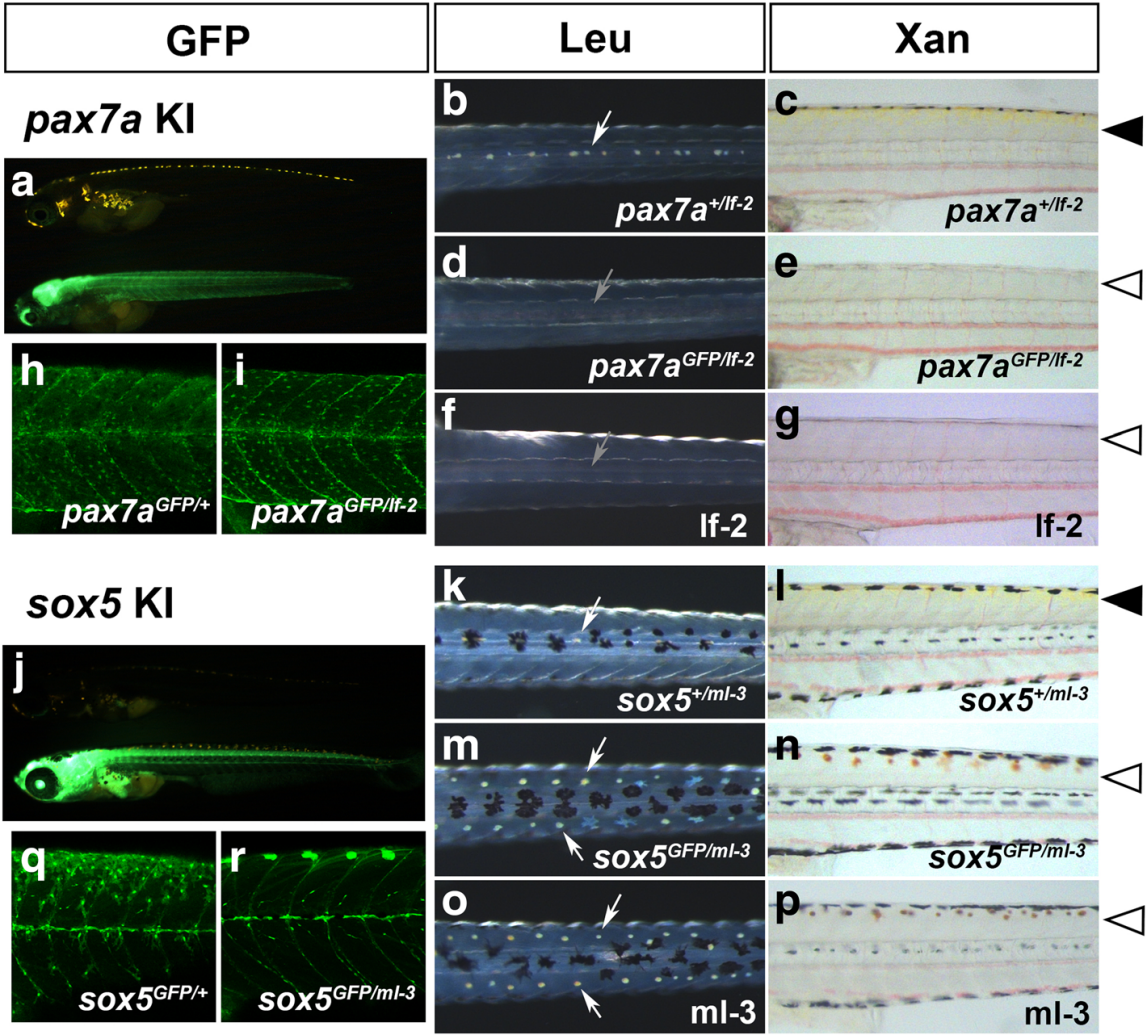
Disruption of gene function by knocking-in *pax7a* and *sox5.* (**a**-**e**, **h**-**i**) Progenies (9 dpf) of a pax7a-transgenic founder crossed to an existing pax7a mutant, leucophore free 2 (lf-2, **f**-**g**). (**j**-**n**, **q**-**r**) Progenies (9 dpf) of a sox5-transgenic founder crossed to an existing sox mutant, many leucophores 3 (ml-3, **o**-**p**). (**a**, **h**, **i**, **j**, **q**, **r**) GFP. (**b**, **d**, **f**, **k**, **m**, **o**) Leucophores viewed in reflected light. (**c**, **e**, **g**, **l**, **n**, **p**) Xanthophores viewed in transmitted light. (**b**, **d**, **f**, **k**, **m**, **o**) Dorsal views of the trunk. (**c**, **e**, **g**, **h**, **i**, **l**, **n**, **p**, **q**, **r**) Lateral views of the trunk. (**h**–**i**, **q**–**r**) Confocal microscopy of the body surface of the nine dpf progenies. (**a**, **j**) Lateral views of pax7a-transgenic fish (**a**) and sox5-transgenic fish (**j**). A non-GFP (upper) and a GFP-positive (lower) larva are shown. Whereas the non-GFP larva (*pax7a*^*+/lf-2*^) has leucophores in the dorsal midline (**b**, white arrow) and shows yellow pigmentation in the skin (**c**, arrowhead), presumably having xanthophores developing normally, the GFP-positive knock-in larva (*pax7a*^*GFP/lf-2*^) fails to have leucophores (**d**, grey arrow) or show yellow pigmentation (**e**, open arrowhead). An lf-2 larva shows the *pax7a* loss-of-function phenotypes (**f**, **g**). Whether having a pax7a knock-in GFP allele with a wild-type allele (**h**, heterozygote) or with an lf-2 allele (**i**, homozygote), the larva has GFP/pax7a-positive progenitors on the surface of the trunk. As shown in B and C, the non-GFP offspring obtained from the sox5-transgenic founder shows normal pigmentation (**k**, white arrow; **l**, arrowhead). The GFP-positive larva has excess leucophores bilaterally along the dorsal midline (**m**, arrows) and lacks yellow pigmentation (**n**, open arrowhead), as does the ml-3 mutant (**o**, **p**). While *sox5* heterozygous larva (**q**, *sox5*^*GFP/+*^) has GFP-positive presumable xanthophore progenitors on the surface of the trunk, *sox5* homozygote (**r**, *sox5*^*GFP/ml-3*^) lacks GFP-positive cells in the corresponding area

We investigated whether the insertion led to disruption of the gene function. To do this, we crossed one of the transgenic founders to a homozygous mutant of *pax7a* or *sox5*, which has defects in skin pigmentation: the *pax7a* homozygotes lack yellow xanthophores and white/orange leucophores, and the *sox5* homozygotes exhibit an absence of xanthophores and excessive formation of leucophores. We found that all of the progeny fish with GFP expression showed the phenotypes of the mutants deficient for the corresponding targeted gene, *pax7a* (Fig. 5a–g) or *sox5* (Fig. 5j–p). The GFP-positive *pax7a* trans-heterozygous larvae (*pax7a*^*GFP/lf-2*^) lacked xanthophores and leucophores (Fig. 5d–e), phenocopying the *pax7a* mutant *lf-2* (Fig. 5f–g). These larvae had GFP-positive cells in the skin (presumably xanthophore progenitors and leucophore) (Fig. 5i), as was seen in *pax7a* heterozygotes (*pax7a*^*GFP/+*^, Fig. 5h), suggesting that *pax7a* function is not required for the formation of the GFP-positive bipotent progenitors, but is for the differentiation of xanthophores and leucophores. Likewise, GFP-positive larvae (trans-heterozygous for GFP insertion and the *sox5* mutation) had excess leucophores and lacked xanthophores (Fig. 5m–n), phenocopying the *sox5* mutant *ml-3* (Fig. 5o-p). The GFP-positive cells were almost completely absent from the dorsal surface of the trunk in *sox5*^*GFP/ml-3*^ larvae (Fig. 5r; compare with wild-type in 5q), suggesting that *sox5* is required for the formation of GFP-positive xanthophore precursors. Non-GFP fish showed essentially normal pigmentation, although we occasionally observed non-GFP fish with the mutant phenotypes. This could be due to the induction of independent indels (induced by CRISPR/Cas9) in the *sox5* locus in different germ cells of the founders. In any case, the results indicate that the insertion of the transgene led to disruption of the gene function.

**Fig. 6:**
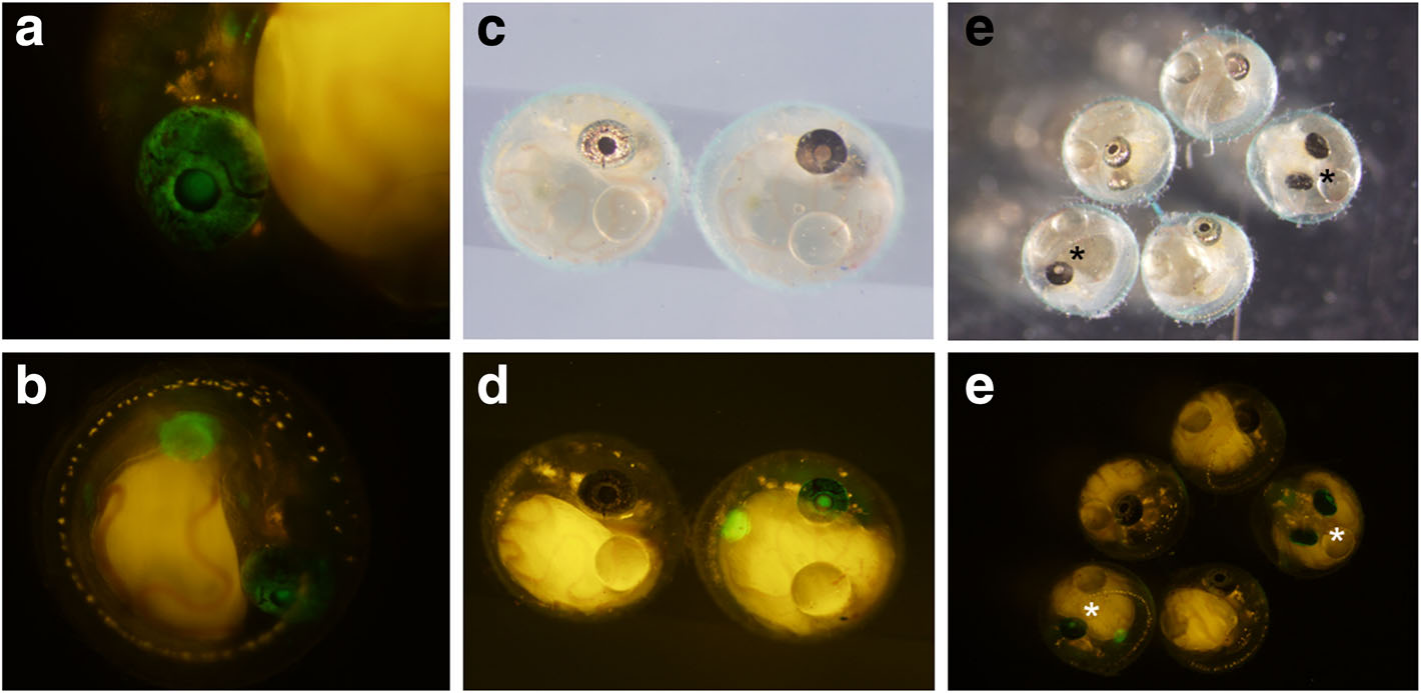
Disruption of gene function by knocking-in *pnp4a****.*** (**a**, **b**) The expression pattern of GFP in founder fish. GFP expression was observed in the eye and abdomen. These fish were considered to have “good expression” of GFP, and were raised to adulthood. GFP expression was observed in the eye and abdomen. (**c**–**f**) The progenies (4 dpf) of a pnp4a-transgenic founder crossed to an existing *gu* mutant. All GFP-positive F1 fish have less-pigmented eyes and all GFP-negative F1 fish have wild-type pigmentation. Asterisks indicate GFP-positive and less-pigmented F1 fish

Next, we performed experiments in another gene, *guanineless (gu)*/*pnp4a*. Mutations of this gene are known to result in defects of guanine formation in iridophores and to result in less-pigmented eyes and abdomens. *pnp4a* expression has been observed in the eye and abdomen at 3–4 dpf with in situ hybridization [19]. As was done in *pax7a* and *sox5*, the target site was set between the transcription start site and the first methionine (Table 1). We performed microinjections to the d-rR strain, which is wild-type at the *gu*/*pnp4a* locus, and screened for fish with good expressions of GFP (Table 2 and Fig. 6a, b). These fish were raised to adulthood, and three transgenic founders (producing GFP-positive F1 fish) were identified out of six fish. The expression patterns of GFP in F1 fish were generally similar across strains, and donor plasmid was knocked-in at the expected site in all founder fish (Additional file 2: Table S2). One GFP-positive founder fish was crossed with homozygous *gu/gu* mutant, which has a deletion of exon 4–7 at the *pnp4a* locus [19]. Among the F1 progenies obtained, GFP-positive fish showed less pigmented iridophores in the eye (right-side embryo in Fig. 6c, d) and GFP-negative fish showed the wild-type phenotype as expected (left-side embryo in Fig. 6c, d). Among the offspring obtained, 16 GFP-positive offspring had the less-pigmented phenotype (*guanineless* phenotype) and 10 GFP-negative offspring had the wild-type phenotype (Fig. 6e, f). These data indicate that the donor plasmid was knocked into the *gu*/*pnp4a* locus and induced a loss of function mutation at the *gu*/*pnp4a* locus.

## Discussion

### Efficient generation of transgenic zebrafish with CRISPR/Cas9

In zebrafish, we have previously shown that knock-in transgenic fish can be efficiently generated via NHEJ by co-injection of two sgRNAs (one for the digestion of the genome and the other for the digestion of donor plasmid), donor plasmid, and Cas9 mRNA. Here, we have shown that the same method is perfectly applicable in medaka. Injected animals frequently show reporter gene expression broadly in cells where targeted genes are expressed (frequency, 30–50% for three of the five genes (*vacht*, *sox5*, and *pax7a*) and 10–20% for the other two genes (*nr5a1* and *pnp4a*); in total, 27% (47 of 177); Table 2). By raising those animals to adulthood, we were able to obtain transgenic founders with very high frequency. Efficiencies exceeded 50% for all five genes (Table 2). Thus, we have succeeded in establishing a highly-efficient knock-in system in medaka fish.

The frequency obtained in this study was higher than that in zebrafish where 5–10% of injected animals usually show “good expression”, and approximately 30% of raised animals became positive founders [6]. One potential reason that accounts for these higher frequencies is the slower development of medaka embryos compared to zebrafish embryos [20, 21]: In medaka, there may be more opportunities for the donor plasmid to integrate in the very early developmental stages.

The NHEJ-mediated knock-in method described here has one clear advantage over the homology-based recombination technique that was recently described by [5]. The current method does not require any DNA construction experiments. By contrast, recombination techniques based on long-homology arms require DNA construction steps. One clear disadvantage of the NHEJ-mediated knock-in system is that it is not suitable for precise knock-in. As shown in Additional file 4: Figure S2, indels were frequently introduced in the joint region, similar to the previous study [18]. For experiments in which precise knock-in is critical, methods that rely on homology-dependent repair systems (i.e., [5]) need to be employed.

### Generation of mutant alleles with NHEJ-mediated knock-in

In this study, we demonstrated that mutant alleles can be efficiently generated by NHEJ-mediated knock-in. For all three of the genes tested, we succeeded in obtaining mutant alleles with high frequency (Table 2). The basic experimental design was that the reporter gene construct (Tbait-hs-GFP) was targeted to be introduced between the transcription start site and the first methionine. This method has several advantages compared to conventional knock-out methods of introducing indels. First, the evaluation of sgRNAs can be achieved readily by screening reporter gene expression in injected animals. The time-consuming molecular biology steps, including DNA extraction, PCR reaction, and sequencing, are not needed. Strikingly, in the present study, we tried only one sgRNA for each of the genes (*sox5*, *pax7a*, and *pnp4a*) and obtained good results in all cases (Table 2). Second, upon establishment of mutant alleles, the strains express a reporter gene in the expression domains of the gene of interest. This makes it possible to monitor the morphological defects of the affected tissues by the expression of the reporter gene. Third, the presence of the reporter gene is greatly beneficial for selecting animals that will inherit the mutant allele in future generations.

### Usage of the hsp70 promoter

As we did in zebrafish, we knocked in constructs with the hsp70 promoter (Figs. 1b and 4a). For this, we extracted a 0.8 kb sequence from the promoter of the medaka *hsp70.1* gene [8]. We showed in this study that this fragment works well as a basal (minimal) promoter. There are several advantages to utilizing the hsp70 promoter in DNA constructs for knock-in. First, the efficiencies of obtaining transgenic founders are likely to be increased, as reporter gene expression occurs irrespective of the direction of integration. Indeed, we found both types of integration (including multi-copy integrations) in the transgenic lines generated in this study. Second, the reporter construct can be targeted virtually anywhere near or within a gene of interest. This feature gives researchers flexibility in designing sgRNAs.

There are two potential disadvantages or concerns regarding the usage of the hsp70 promoter. First, the promoter has activity in female germ cells. We observed maternally derived fluorescence in many of the lines generated in this study. Although this did not present major problems in the present study, it could be a problem for monitoring the expression patterns of genes in the early stages. Nonetheless, this problem can likely be overcome, in many cases, by using transgenic males for mating. Second, gene expression may not be completely recapitulated with the usage of a heterologous promoter. Although reporter gene expressions appeared to mimic the expressions of the endogenous genes in the transgenic fish generated in the current study for all five of the genes, the possibility of an occurrence of ectopic expressions cannot be completely excluded. Indeed, we have noted an ectopic expression in a Tg[evx2-hs:Gal4] transgenic fish strain in the case of zebrafish [6]. Thus, researchers need to be aware of the potential occurrence of an ectopic expression when using the hsp70 promoter.

### Conversion of reporter gene expression by the Cre-loxP system

In this study, we showed that RFP expression can be changed to GFP expression in transgenic fish with the loxP-RFP-loxP-GFP construct. To make this occur ubiquitously, we established Tg[zhspa8:Cre-mCherry-NLS] in which the Cre-mCherry-NLS fusion protein is expressed ubiquitously in early embryos. Using this line, one reporter gene was easily converted to another reporter/driver gene by crossing alone. This is a powerful system for establishing transgenic fish that express a reporter/driver gene that is itself not fluorescent (without the aid of fluorescent reporters, prescreening before raising animals is not possible). For example, we succeeded in establishing several Gal4 driver lines using this system in zebrafish (our unpublished observation). In these studies, we first established loxP-RFP-loxP-Gal4 lines, and RFP was then converted to Gal4 by utilizing Tg[zhspa8:Cre-mCherry-NLS] transgenic zebrafish. The same strategy could be used to generate transgenic medaka that express reporter/driver genes that are not fluorescent themselves.

## Conclusion

We report that the NHEJ-mediated knock-in system is highly efficient in medaka, and is very useful for establishing mutant alleles. With its simplicity and high efficiency, we propose that the method described may become a standard technique for the generation of transgenic and mutant medaka.

## Additional files

Additional file 1: Table S1. Genomic location of potential off-target sites for sgT (sgRNA for Tbait) (DOCX 14 kb)

Additional file 2: Table S2. Status of the insertions in the transgenic fish generated in this study (DOCX 16 kb)

Additional file 3: Figure S1. GFP expression in Tg[pax7a-hs:GFP] and Tg[sox5-hs:GFP]. (A, B) Tg[pax7a-hs:GFP]. (C, D) Tg[sox5-hs:GFP]. (A, C) Dorsal views of the head region magnified. (B, D) Lateral views of the anterior trunk region magnified. The signal in the tectum is especially strong in Tg[pax7a-hs:GFP] (A). Presumable pigment cell progenitors of xanthophore and leucophore (B) and xanthophore (D) on the body surface are positive for GFP in Tg[pax7a-hs:GFP] and Tg[sox5-hs:GFP], respectively. (JPEG 1442 kb)

Additional file 4: Figure S2. Nucleotide sequences of the joint region of the insertions for the sox5 and pax7a transgenic fish The PCR products that span the genomic DNA and the donor DNA (see Additional file 2: Table S2) were sequenced. The top two lines for each panel show the sequence of the genome and the donor. Underlined sequences correspond to sgRNA targets. The PAM sequences are indicated in green. The predicted digestion sites by CRISPR/Cas9 are indicated with arrowheads. Sequences in bold letters are expected to be present after the integrations. The predicted nucleotide sequence without an indel is shown in the third line as bold letters. (A) The 5′ side of the *sox5* transgenic fish with the forward integration (#1, #3, and #4 strains; Additional file 2:Table S2). (B) The 5′ side of the *sox5* transgenic fish with the reverse integration (#2 strains; Additional file 2: Table S2). (C) The 3′ side of the *sox5* transgenic fish with the forward integration (#1, #2, #3, and #7 strains; Additional file 2: Table S2). (D) The 3′ side of the *sox5* transgenic fish with the reverse integration (#6 strain; Additional file 2: Table S2). (E) The 5′ side of the *pax7a* transgenic fish with the forward integration (#1, #3, #4, and #6 strains; Additional file 2: Table S2). (F) The 5′ side of the *pax7a* transgenic fish with the reverse integration (#2 strains; Additional file 2: Table S2). (G) The 3′ side of the *pax7a* transgenic fish with the forward integration (#1and #6 strains; Additional file 2: Table S2). (H) The 3′ side of the *pax7a* transgenic fish with the reverse integration (#2, #3, #4, and #5 strains; Additional file 2: Table S2). (PDF 31 kb)
