# Supplementary material for: Highly efficient generation of knock-in transgenic medaka by CRISPR/Cas9-mediated genome engineering
Source: Zoological Lett. 2018 Feb 5;4:3. doi: 10.1186/s40851-017-0086-3 (PMC5798193; doi:10.1186/s40851-017-0086-3)
Supplement: Supplementary file 1 — Genomic location of potential off-target sites for sgT (sgRNA for Tbait) (DOCX 14 kb) [file 40851_2017_86_MOESM1_ESM.docx]

Table S1

| chromosome | start(bp) | end(bp) | strand | matching pattern |
| --- | --- | --- | --- | --- |
| 13 | 25214193 | 25214171 | reverse | GGCTGCTGcgAtGGAGCTCAAGG |
| 15 | 10654407 | 10654385 | reverse | tGCTGCTGTCAGtGAGCgCACGG |
