# Supplementary material for: Highly efficient generation of knock-in transgenic medaka by CRISPR/Cas9-mediated genome engineering
Source: Zoological Lett. 2018 Feb 5;4:3. doi: 10.1186/s40851-017-0086-3 (PMC5798193; doi:10.1186/s40851-017-0086-3)
Supplement: Supplementary file 2 — Status of the insertions in the transgenic fish generated in this study (DOCX 16 kb) [file 40851_2017_86_MOESM2_ESM.docx]

TableS2

|  | founder | 5’ | 3’ | tandem with the same direction |
| --- | --- | --- | --- | --- |
| vacht | #1 | N.A.^1^ | forward | - |
|  | #2 | N.A.^1^ | forward | + |
|  | #3 | N.A.^1^ | reverse | - |
|  | #4 | N.A.^1^ | reverse | - |
|  | #5 | N.A.^1^ | reverse | + |
| nr5a1 | #1 | forward | forward | - |
| sox5 | #1 | forward | forward | + |
|  | #2 | reverse | forward | - |
|  | #3 | forward | forward | + |
|  | #4 | forward | N.D. | + |
|  | #5 | N.D. | N.D. | + |
|  | #6 | N.D. | reverse | - |
|  | #7 | N.D. | forward | + |
| pax7a | #1 | forward | forward | - |
|  | #2 | reverse | reverse | - |
|  | #3 | forward | reverse | + |
|  | #4 | forward | reverse | + |
|  | #5 | N.D. | reverse | - |
|  | #6 | forward | forward | - |
| pnp4a | #1 | forward | forward | - |
|  | #2 | forward | forward | N.A.^2^ |
|  | #3 | forward | N.D. | N.A.^2^ |
