# Supplementary figures and images for: Highly efficient generation of knock-in transgenic medaka by CRISPR/Cas9-mediated genome engineering
Source: Zoological Lett. 2018 Feb 5;4:3. doi: 10.1186/s40851-017-0086-3 (PMC5798193; doi:10.1186/s40851-017-0086-3)

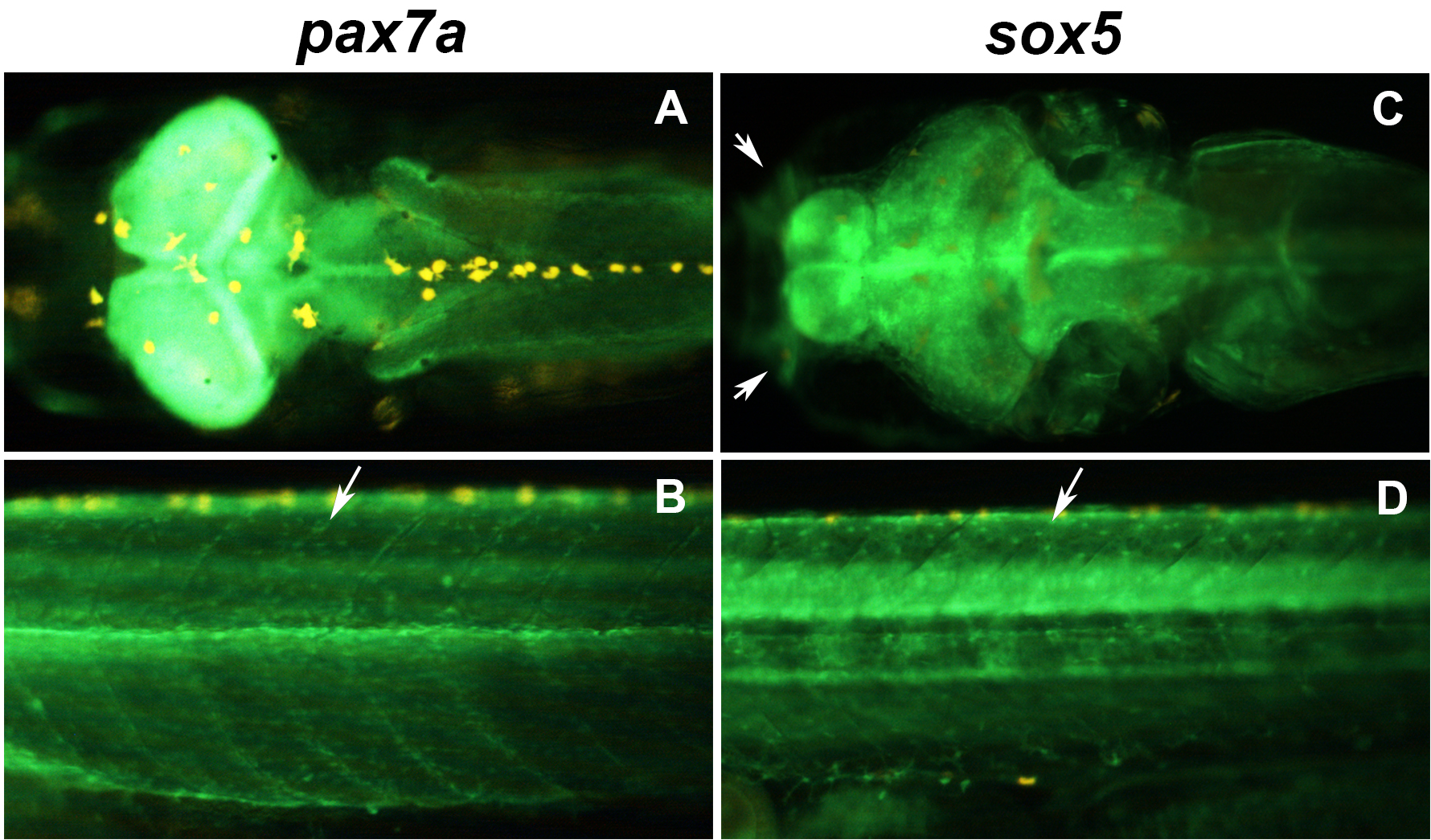

Supplement: Supplementary file 3 — GFP expression in Tg[pax7a-hs:GFP] and Tg[sox5-hs:GFP]. (A, B) Tg[pax7a-hs:GFP]. (C, D) Tg[sox5-hs:GFP]. (A, C) Dorsal views of the head region magnified. (B, D) Lateral views of the anterior trunk region magnified. The signal in the tectum is especially strong in Tg[pax7a-hs:GFP] (A). Presumable pigment cell progenitors of xanthophore and leucophore (B) and xanthophore (D) on the body surface are positive for GFP in Tg[pax7a-hs:GFP] and Tg[sox5-hs:GFP], respectively. (JPEG 1442 kb) [file 40851_2017_86_MOESM3_ESM.jpg]
