# Supplementary material for: Highly efficient generation of knock-in transgenic medaka by CRISPR/Cas9-mediated genome engineering
Source: Zoological Lett. 2018 Feb 5;4:3. doi: 10.1186/s40851-017-0086-3 (PMC5798193; doi:10.1186/s40851-017-0086-3)
Supplement: Supplementary file 4 — Nucleotide sequences of the joint region of the insertions for the sox5 and pax7a transgenic fish The PCR products that span the genomic DNA and the donor DNA (see Additional file 2: Table S2) were sequenced. The top two lines for each panel show the sequence of the genome and the donor. Underlined sequences correspond to sgRNA targets. The PAM sequences are indicated in green. The predicted digestion sites by CRISPR/Cas9 are indicated with arrowheads. Sequences in bold letters are expected to be present after the integrations. The predicted nucleotide sequence without an indel is shown in the third line as bold letters. (A) The 5′ side of the sox5 transgenic fish with the forward integration (#1, #3, and #4 strains; Additional file 2:Table S2). (B) The 5′ side of the sox5 transgenic fish with the reverse integration (#2 strains; Additional file 2: Table S2). (C) The 3′ side of the sox5 transgenic fish with the forward integration (#1, #2, #3, and #7 strains; Additional file 2: Table S2). (D) The 3′ side of the sox5 transgenic fish with the reverse integration (#6 strain; Additional file 2: Table S2). (E) The 5′ side of the pax7a transgenic fish with the forward integration (#1, #3, #4, and #6 strains; Additional file 2: Table S2). (F) The 5′ side of the pax7a transgenic fish with the reverse integration (#2 strains; Additional file 2: Table S2). (G) The 3′ side of the pax7a transgenic fish with the forward integration (#1and #6 strains; Additional file 2: Table S2). (H) The 3′ side of the pax7a transgenic fish with the reverse integration (#2, #3, #4, and #5 strains; Additional file 2: Table S2). (PDF 31 kb) [file 40851_2017_86_MOESM4_ESM.pdf]

A sox5 5’ side, forward integration

```
sox5 genome              5′ GGTGGGGAGACTGAGCGCCGCTTCGGCTGTCCGCGCGCGA 3′
donor plasmid            5′ CGCGGCTGCTGTCAGGGAGCTCATGGAGCTGCGTCACGTG 3′
                                               ↓
predicted sequence
  without an indel       5′ GGTGGGGAGACTGAGCGCCGTCATGGAGCTGCGTCACGTG 3′

sox5 #1                  5′ GGTGGGGAGACTGAGCG--------------CGTCACGTG 3′
sox5 #3                  5′ GGTGGGGAGACTGAGCG--------------CGTCACGTG 3′
sox5 #4                  5′ GGTGGGGAGACTGAGCGCCG-------GCTGCGTCACGTG 3′
                                              GTTCTG
```

B sox5 5’ side, reverse integration

```
sox5 genome              5′ GGTGGGGAGACTGAGCGCCGCTTCGGCTGTCCGCGCGCGA 3′
donor plasmid            5′ CACGTGACGCAGCTCCATGAGCTCCCTGACAGCAGCCGCG 3′
                                               ↓
predicted sequence
  without an indel       5′ GGTGGGGAGACTGAGCGCCGGCTCCCTGACAGCAGCCGCG 3′

sox5 #2                  5′ GGTGGGGAGACTGA-----------------GCAGCCGCG 3′
                                                 CG
```

C sox5 3’ side, forward integration

```
sox5 genome              5′ GGTGGGGAGACTGAGCGCCGCTTCGGCTGTCCGCGCGCGA 3′
donor plasmid            5′ CGCGGCTGCTGTCAGGGAGCTCATGGAGCTGCGTCACGTG 3′
                                               ↓
predicted sequence
  without an indel       5′ CGCGGCTGCTGTCAGGGAGCCTTCGGCTGTCCGCGCGCGA 3′

sox5 #1                  5′ CGCG----------------CTTCGGCTGTCCGCGCGCGA 3′
                              AATGAATAACGGTTTGGTTGATGCGAGTGATTTCT
sox5 #2                  5′ CGCGGCTGCTGTCAG-----CTTCGGCTGTCCGCGCGCGA 3′
                                            CCGTTTC
sox5 #3                  5′ -----------------------CGGCTGTCCGCGCGCGA 3′
                                   GCCGCGAACTGGTCAGTTCAGTTCG
sox5 #7                  5′ CGCGGCTGCTGTC-------------CTGTCCGCGCGCGA 3′
                                  GTGATTGCGCCTGAGCGAGACGAAATA
```

D sox5 3’ side, reverse integration

```
sox5 genome              5′ GGTGGGGAGACTGAGCGCCGCTTCGGCTGTCCGCGCGCGA 3′
donor plasmid            5′ CACGTGACGCAGCTCCATGAGCTCCCTGACAGCAGCCGCG 3′
                                               ↓
predicted sequence
  without an indel       5′ CACGTGACGCAGCTCCATGACTTCGGCTGTCCGCGCGCGA 3′

sox5 #6                  5′ CACGTGACGCAGC----------CGGCTGTCCGCGCGCGA 3′
                                      GTAAAACATATCAA
```

E pax7a 5’ side, forward integration

```
pax7a genome                    5′ TGAAGGCTCGATCAGTGTCCAGTTGGGTGTTTTTGCCTGG 3′
donor plasmid                   5′ CGCGGCTGCTGTCAGGGAGCTCATGGAGCTGCGTCACGTG 3′
                                                       ↓
predicted sequence
  without an indel              5′ TGAAGGCTCGATCAGTGTCCTCATGGAGCTGCGTCACGTG 3′

pax7a #1                        5′ TGAAGGCTCGATCAGTGTCCTCATGGAGCTGCGTCACGTG 3′
pax7a #3                        5′ TGAAGGCTCGATCAGTG---TCATGGAGCTGCGTCACGTG 3′
pax7a #4                        5′ TGAAGGCTCGATCAGTG---TCATGGAGCTGCGTCACGTG 3′
pax7a #6                        5′ TGAAGGCTCGATCA------TCATGGAGCTGCGTCACGTG 3′
```

F pax7a 5’ side, reverse integration

```
pax7a genome                    5′ TGAAGGCTCGATCAGTGTCCAGTTGGGTGTTTTTGCCTGG 3′
donor plasmid                   5′ CACGTGACGCAGCTCCATGAGCTCCCTGACAGCAGCCGCG 3′
                                                       ↓
predicted sequence
  without an indel              5′ TGAAGGCTCGATCAGTGTCCGCTCCCTGACAGCAGCCGCG 3′

pax7a #2                        5′ TGAAGGCTCGATCAGTGTCCGCTCCCTGACAGCAGCCGCG 3′
```

G pax7a 3’ side, forward integration

```
pax7a genome                    5′ TGAAGGCTCGATCAGTGTCCAGTTGGGTGTTTTTGCCTGG 3′
donor plasmid                   5′ CGCGGCTGCTGTCAGGGAGCTCATGGAGCTGCGTCACGTG 3′
                                                       ↓
predicted sequence
  without an indel              5′ CGCGGCTGCTGTCAGGGAGCAGTTGGGTGTTTTTGCCTGG 3′

pax7a  #1                       5′ CGCGGCTGC------------GTTGGGTGTTTTTGCCTGG 3′
                                                       G
pax7a  #6                       5′ CGCGGCTGCTGTCAGGGAGCAGTTGGGTGTTTTTGCCTGG 3′
```

H pax7a 3’ side, reverse integration

```
pax7a genome                    5′ TGAAGGCTCGATCAGTGTCCAGTTGGGTGTTTTTGCCTGG 3′
donor plasmid                   5′ CACGTGACGCAGCTCCATGAGCTCCCTGACAGCAGCCGCG 3′
                                                       ↓
predicted sequence
  without an indel              5′ CACGTGACGCAGCTCCATGAAGTTGGGTGTTTTTGCCTGG 3′

pax7a #2                        5′ CACGTGACGCAGCTCCAT---GTTGGGTGTTTTTGCCTGG 3′
pax7a #3                        5′ CACGTGACGCAGCTCCATG---TTGGGTGTTTTTGCCTGG 3′
                                                    TTGGGTGT
pax7a #4                        5′ CACGTGACGCAGCTCCATG---TTGGGTGTTTTTGCCTGG 3′
                                                    TTGGGTGT
pax7a #5                        5′ CACGTGACGCAGCTC-----AGTTGGGTGTTTTTGCCTGG 3′
```
